# Determination of Magnesium Valproate and Its Process Related Impurities by Ultraperformance Liquid Chromatography

**DOI:** 10.1155/2014/412704

**Published:** 2014-07-14

**Authors:** Rakshit Thakkar, Hitesh Saravaia, Madhavi Patel, Anamik Shah

**Affiliations:** National Facility for Drug Discovery through NCEs Development & Instrumentation Support to SMPEs, Department of Chemistry, Saurashtra University, Rajkot, Gujarat 360005, India

## Abstract

A selective ultraperformance liquid chromatographic (UPLC) method for the determination of magnesium valproate and its process related impurities has been developed. The method includes reversed-phase Acquity BEH C_18_ column with 100 mm × 2.1 mm i.d. and 1.7 *µ* particle size. The mobile phase consists of acetonitrile and 5 mM ammonium dihydrogen orthophosphate with pH = 3.0 at 45 : 55 isocratic elution. The flow rate was set at 0.3 mL/min and UV detection was performed at 215 nm. A system suitability test (SST) was developed to govern the quality of the separation. The developed method has been validated further with respect to linearity, accuracy, precision, selectivity, LOD, LOQ, and robustness. Different batches of samples were examined using this method; the method proved to be successful when applied to analyze a marketed magnesium valproate formulation.

## 1. Introduction

**Figure 1:**
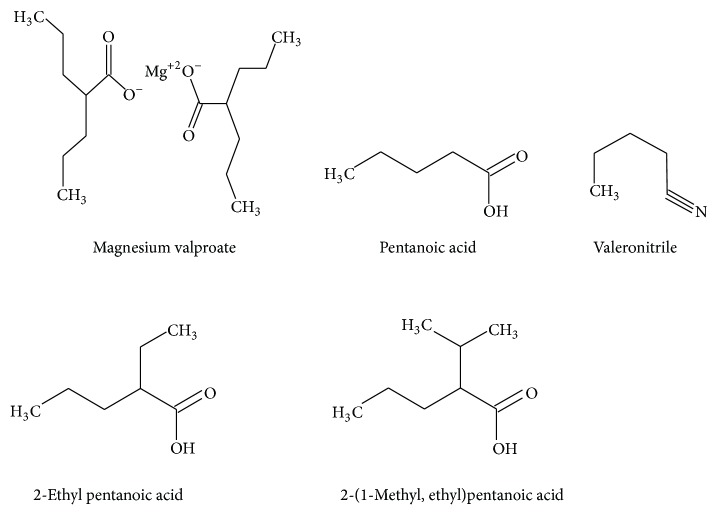
Chemical structure of magnesium valproate and its process related impurities.

Magnesium valproate is chemically known as magnesium 2-propylpentanoate. Magnesium valproate is an anticonvulsant used in the treatment of epilepsy and bipolar disorder, as well as other psychiatric conditions requiring the administration of a mood stabilizer [1–6]. Chemical structures of magnesium valproate and four of its process related impurities are given in Figure 1. To our knowledge, there is no paper describing an ultraperformance liquid chromatographic (UPLC) method that allows the separation of magnesium valproate and its impurities in bulk drugs. Some articles exist on isocratic liquid chromatographic methods for the determination of magnesium valproate [7, 8]. However, these isocratic methods use short columns and are suitable for assay only since they focus on the main peak. The gas chromatographic methods and colorimetric methods are also in literature [9–11]. The methods for determination of valproic acid and sodium valproate are also useful for achieving the best results in this work [12–14]. Some of the bioanalytical methods are also available in literature [15–21]. Applying the above methods for the separation of impurities in a bulk magnesium valproate sample gives poor separation. Therefore, an attempt was made to develop a new, rapid, and sensitive method for the determination of magnesium valproate and its process related impurities. To access the reproducibility and wide applicability of the developed method, it was validated as per international code of harmonization norm, which is also mandatory [22]. This paper also deals with the validation of the developed UPLC method for the assay of magnesium valproate from its bulk and pharmaceutical dosage form.

## 2. Material and Methods

### 2.1. Chemicals and Reagents

Parth Laboratories Pvt. Ltd. (Rajkot, India) has provided magnesium valproate and its process related impurities working standards and samples. Ammonium dihydrogen orthophosphate for HPLC, orthophosphoric acid (HPLC grade), and HPLC grade acetonitrile were purchased from Spectrochem Pvt. Ltd. HPLC grade water used was purified by Milli-Q Elix-3 water purification system.

### 2.2. Instrumentation

The Waters Acquity UPLC chromatographic system was used to perform development and validation. This system consists of a binary solvent manager, multiple wavelength ultraviolet detector, sample manager, and column oven connected to a multi-instrument data acquisition and processing system Empower 2.1 version. Sartorius microbalance and Equiptronics branded balance and heating oven was used for the weighing and heating purpose while Spinco ultrasonic bath was used for degassing purpose.

### 2.3. Method Development

Analytical method development consists of the following steps which can be bound up by the literature survey, previous experience, and chemical nature of the reagents used in the development.

### 2.4. Mobile Phase Selection

On the basis of literature survey, several exploratory runs have been performed but initially proper selectivity and resolution between the drug substance and its impurities were not achieved. After furnishing more importance to the literature, it was concluded that since one impurity specifically 2-(1-ethyl-methyl)pentanoic acid is a structural isomer of the drug component (valproic acid), the 5 mM ammonium dihydrogen orthophosphate with pH = 3.0 has given the maximum resolution with acetonitrile as organic component at isocratic elution, 55 : 45, v/v.

### 2.5. Column Selection

Column selection is the most important part in the method development. In this case most suitable column chemistry was bridge ethyl hybrid (BEH) C_18_ among the column chemistry available with us. Acquity BEH C_18_ (100 mm × 2.1 mm i.d., 1.7 *μ* particle size) has given the best outcomes. The resolution between the 2-(1-ethyl-methyl)pentanoic acid and the valproate salt was not achieved by the other columns.

### 2.6. Detection Wavelength Selection

The standard solution was screened over 190 nm to 400 nm using the advantage of photodiode array detector. On the basis of peak absorption maxima and peak purity index, the 215 nm was decided as the detection wavelength which has provided the maximum chromatographic compatibility to the method.

### 2.7. Mobile Phase Preparation

The mobile phase consists of acetonitrile: 5 mM ammonium dihydrogen orthophosphate (pH = 3.0) (45 : 55) was prepared by dissolving 575 milligram of ammonium dihydrogen orthophosphate for HPLC in 1000 mL ultrapure (HPLC grade) water; then the pH of the buffer solution was adjusted up to 3.0 using 0.1% orthophosphoric acid solution. This was further mixed with acetonitrile by mentioned ratio and filtered through 0.22 *μ*n filter followed by degassing in ultrasonic bath for 20 min.

### 2.8. Standard Solution Preparation

**Figure 2:**
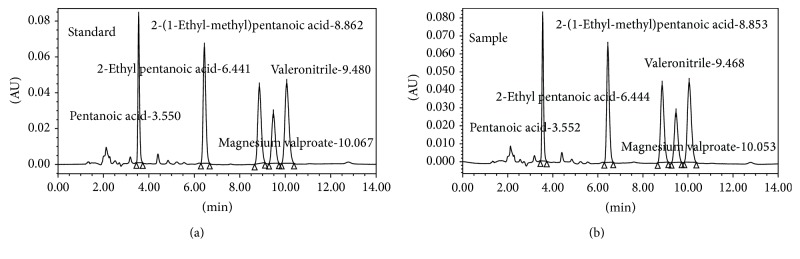
Chromatograms of working standard and sample solution.

Magnesium valproate and its related impurities working standard solution containing 200 *μ*g/mL were prepared in a 100 mL volumetric flask by dissolving 20.0 mg each in 25 mL acetonitrile : water (50 : 50) and then diluting to volume with the same diluent. Further this was filtered through 0.22 *μ*n filter followed by degassing in ultrasonic bath for 20 min. The chromatogram of working standard is given in Figure 2.

### 2.9. Sample Solution Preparation

**Figure 3:**
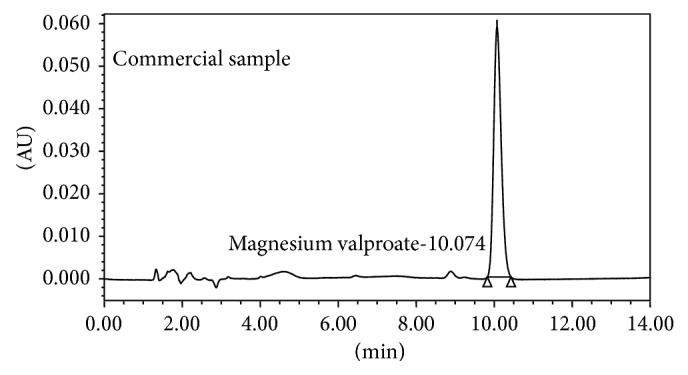
Chromatogram of commercial sample of magnesium valproate.

Magnesium valproate and its related impurities sample (different batch) solution containing 200 *μ*g/mL were prepared in a 100 mL volumetric flask by dissolving 20 mg each in 25 mL acetonitrile : water (50 : 50) and then diluting to volume with the same diluent. Commercially, magnesium valproate is available in 200 mg, 300 mg, and 500 mg pharmaceutical dosage form. Average weight of the tablet was found by weighing 20 tablets and then it was dissolved and diluted to achieve the 200 *μ*g/mL concentration. Further this was filtered through 0.22 *μ*n filter followed by degassing in ultrasonic bath for 20 min. The chromatogram of sample solution and commercial is shown in Figures 2 and 3, respectively.

## 3. Results and Discussion

With reference to the method development, chromatographic parameters used for method validation experiments are given in Table 1.

Method for the determination of magnesium valproate and its related impurities in bulk drug is further validated as per ICH Q2(R1) guideline [21]. Validation of analytical method was performed using magnesium valproate and its process related impurities working standard and sample batch drug substance.

The accuracy experiment was performed by recovery study at three levels, 150%, 100%, and 50%, of the standard concentration. The working standard of magnesium valproate and its related impurities were added to the samples and the recovery was calculated which was between 98 and 102%; these were well within the acceptance criteria.

The method precision was assessed using multiple preparations of a single sample. Six different preparations of the same magnesium valproate and its related impurities sample, each 0.20 mg/mL, were analyzed in triplicate on the same day. New solutions were prepared and analyzed on each of two successive days. The %RSD values obtained for the peak areas of magnesium valproate, valeronitrile, pentanoic acid, 2-ethyl pentanoic acid, and 2-(1-methyl, ethyl)pentanoic acid were not more than 2.0. The intermediate precision study was performed using another Acquity BEH C_18_ column (100 mm × 2.1 mm i.d., 1.7 *μ* particle size). The %RSD values were of the same magnitude as above.

The linearity of the method was evaluated by analyzing eight solutions in the concentration ranges 50–350 *μ*g/mL for each solution of magnesium valproate and its related impurities. The peak areas obtained from different concentrations of the drugs were used to calculate linear regression equations. These were *y* = 10.15*x* − 4.714, *y* = 10.06*x* + 21, *y* = 10.12*x* + 5.285, and *y* = 10.37*x* − 21.85 with correlation coefficients of *R* ^2^ = 0.999, *R* ^2^ = 0.998, *R* ^2^ = 0.999, *R* ^2^ = 0.997, and *R*² = 0.998 for pentanoic acid, 2-ethyl pentanoic acid, 2-(1-methyl, ethyl)pentanoic acid, valeronitrile, and magnesium valproate, respectively. The high values of the correlation coefficients were indicative of linear relationships between analyte concentration and peak area.

The limits of detection (LOD) and quantification (LOQ) were established by evaluating the minimum level at which the analytes could be readily detected and quantified with accuracy, respectively. The LOD and LOQ for each component were 1.0 *μ*g/mL and 2.0 *μ*g/mL. The signal to noise ratio was more than 3 and 10 for LOD and LOQ, respectively.

The selectivity of the method was evaluated by injecting blank matrix, each individual impurity, magnesium valproate standard solution, and spiked solution to check the interference of the diluent as well as the standard solution on each other. The method was proved as highly selective that there was no interference on any component to others. The separation factor (*α*) was investigated for all the impurities, which found 0.33, 0.64, 0.88, and 0.90 for pentanoic acid, 2-ethyl pentanoic acid, 2-(1-methyl, ethyl)pentanoic acid, and valeronitrile, respectively.

The influence of five chromatographic parameters (*k*) on the separation was investigated. The parameters examined were the amount of acetonitrile in mobile phase, the pH of the ammonium dihydrogen orthophosphate solution, and the amount of ammonium dihydrogen orthophosphate solution in the mobile phase. No such impact of the small changes on the above parameters was observed which suggest that the method is highly robust.

The developed UPLC method shows a good separation of magnesium valproate to its impurities. The robustness study indicated that mainly the pH of the ammonium dihydrogen orthophosphate solution should be monitored carefully to ensure the best separation as this has a significant effect on the separation. The method is found to be selective, precise, sensitive, and linear, which is also proved from the summary of method validation (Table 2). The method can be used for the determination of magnesium valproate and the identification of the impurities present in the pharmaceutical dosage form.

## Figures and Tables

**Table 1:** Chromatographic conditions of proposed analytical method.

Parameters	Optimum condition
Mobile phase A	5 mM ammonium dihydrogen orthophosphate (PH = 3.0)
Mobile phase B	acetonitrile (HPLC grade)
Column	Acquity BEH C_18_ (100 mm × 2.1 mm i.d., 1.7 *μ* particle size)
Flow rate	0.3 mL/min
Isocratic elution at	55 : 45, v/v
Detection	215 nm UV
Diluent	Acetonitrile : water (1 : 1)
Injection volume	5 *μ*L

**Table 2:** Summary of validation study.

Validation experiment	PA	2-EPA	2-1-MEPA	VA	MgVA
Specificity	No interference	No interference	No interference	No interference	No interference
LOQ (*μ*g/mL)	2.0	2.0	2.0	2.0	2.0
LOD (*μ*g/mL)	1.0	1.0	1.0	1.0	1.0
Linearity					
(I) Correlation coefficient	0.999	0.998	0.999	0.997	0.998
(II) Regression equation	*y* = 10.15*x* − 4.714	*y* = 10.06*x* + 21	*y* = 10.12*x* + 5.285	*y* = 10.37*x * − 21.85	*y* = 10.29*x* − 13.28
Method precision (*n* = 6)	%RSD = 0.2	%RSD = 0.2	%RSD = 0.2	%RSD = 1.2	%RSD = 0.1
Int. precision (*n* = 6)	%RSD = 1.2	%RSD = 0.8	%RSD = 0.8	%RSD = 1.8	%RSD = 0.7
Accuracy (% recovery)	99–101	98–100	99-100	98–102	99-100
Robustness	No significant change	No significant change	No significant change	No significant change	No significant change
